# Melioidosis: An Australian Perspective

**DOI:** 10.3390/tropicalmed3010027

**Published:** 2018-03-01

**Authors:** Simon Smith, Josh Hanson, Bart J. Currie

**Affiliations:** 1 Department of Medicine, Cairns Hospital, Cairns, QLD 4870, Australia; Joshua.Hanson@health.qld.gov.au; 2 James Cook University Clinical School, Cairns Hospital, Cairns, QLD 4870, Australia; 3 Global and Tropical Health Division, Menzies School of Health Research, Charles Darwin University, Darwin, NT 0811, Australia; Bart.Currie@menzies.edu.au; 4 The Kirby Institute, University of New South Wales, Sydney, NSW 2052, Australia; 5 Department of Infectious Diseases, Royal Darwin Hospital, Darwin, NT 0811, Australia

**Keywords:** melioidosis, Australia, tropical medicine

## Abstract

*Burkholderia pseudomallei* is endemic in northern Australia, with cases of melioidosis most commonly occurring during the wet season in individuals with diabetes, hazardous alcohol use, and chronic kidney disease. Pneumonia is the most common presentation and the majority of patients are bacteraemic—however, infection may involve almost any organ, with the skin and soft tissues, genitourinary system, visceral organs, and bone and joints affected most commonly. Central nervous system involvement is rarer, but has a high attributable mortality. Increased awareness of the disease amongst healthcare providers, ready access to appropriate antibiotic therapy and high-quality intensive care services has resulted in a sharp decline in the case fatality rate over the last 20 years. Further improvement in clinical outcomes will require a greater understanding of the disease′s pathophysiology, its optimal management, and more effective strategies for its prevention.

## 1. History

Melioidosis was initially described in Australia in 1949, following an outbreak in sheep in central west Queensland [1]. The first human case of melioidosis in Australia was described in Townsville, North Queensland from 1950 [2] and cases were subsequently reported in the Northern Territory (NT) from 1960 [3]. 

Melioidosis is endemic across northern, tropical Australia north of latitude 20° S [4,5,6,7], with focal areas of endemicity described in much more southern, temperate regions (latitude 31° S) [8,9,10]. Outbreaks, related to contaminated water, have been described in pigs in south-east Queensland (latitude 25.5° S) [11] and human and bovine cases have also been identified in the same region [12,13,14]. Even in dry, arid desert regions of Central Australia, cases of melioidosis have occurred following intense rainfall, highlighting the organism’s ability to survive in harsh environments [15].

The incidence of melioidosis differs across northern Australia. In the Top End of the NT, yearly incidence rates range between 5.4 and 50.2/100,000 population [6,16]. Indigenous Australians are disproportionately affected and bear the greatest burden of the disease. During the monsoonal rains of 2009–2010, a wet season with above average rainfall, incidence increased to 102.4/100,000 population in the Top End Indigenous population [16]. In the Torres Strait Islands, the mean incidence is 33.1/100,000 population [17].

Despite the high incidence of disease in northern Australia, background seropositivity rates are relatively low compared to those seen in Southeast Asia, even if a lower indirect haemagglutination assay titre cut-off of 1:40 is used. Rates range from 5 to 12.8%, with the highest identified in Indigenous Australians and people living in rural locations [18,19].

It was previously believed that *Burkholderia pseudomallei* colonised Australia from Southeast Asia, but phylogeographic reconstruction suggests an Australian origin for *B. pseudomallei*, with dispersal into Southeast Asia occurring after one or more introduction events during the last glacial period [20]. 

*B. pseudomallei* in northern Australia is genetically diverse; there is significant differentiation between the genotypes present in isolates from Queensland and the Northern Territory [21]. The organism rarely moves across major biogeographic boundaries; however, in Darwin, an Asian *B. pseudomallei* strain, sequence type (ST)-562, has become a common ST affecting patients with melioidosis in this area [22].

## 2. Melioidosis Cases and the Presence of *B. pseudomallei*

*B. pseudomallei* has been identified from a number of Australian animals, including goats [23], sheep [1], camels [24], and alpacas [25], all of which are considered to be highly susceptible to melioidosis [26]. Melioidosis commonly presents as mastitis in goats, but zoonotic transmission remains exceedingly rare [26]. Outbreaks have occurred in pigs [11] and cases have been identified in a wide variety of domestic and native Australia animals and birds [27,28,29,30,31,32,33,34]. *B. pseudomallei* has been detected in faecal samples from wallabies and chickens, suggesting that faecal shedding may contribute to the geographical expansion of the disease [35].

Melioidosis in humans is considered an opportunistic infection in Australia, with the vast majority of people having at least one identifiable risk factor. The most common risk factors identified are diabetes mellitus, hazardous alcohol use, chronic lung disease, and chronic kidney disease [6]. In healthy people, death from melioidosis is considered extremely rare if appropriate antibiotics and intensive care support are available [6,17].

In Australia, melioidosis is usually acquired percutaneously or by inhalation. Infection by ingestion is considered unusual, although outbreaks caused by contaminated drinking water supplies have occurred [36,37]. In northern Australia, cases of melioidosis follow a seasonal pattern and are strongly associated with monsoonal rains [17,38,39]. An increase in the dew point, cloud cover, temperature, rainfall, and groundwater have all been associated with an increased risk of the disease [40]. An increase in the number of people with melioidosis pneumonia has been attributed to cyclones and tropical storms in the NT [41] and cyclones coincide with increased melioidosis cases in Western Australia [42]; however, no correlation is seen between melioidosis cases and severe weather events in Far North Queensland (FNQ) [43]. Melioidosis cases associated with severe weather events have been shown to be caused by different *B. pseudomallei* MLST genotypes, suggesting that airborne dissemination may not come from a common source [44].

Pneumonia is the most common presentation of melioidosis in Australia [45]. Bacteraemic presentations vary between 55% and 74% with particularly high rates observed in FNQ [6,17]. While this may partly be explained by differences in case findings, it is yet to be determined if strains in FNQ are intrinsically more virulent.

Genitourinary involvement is common in Australia, with prostate abscesses occurring in up to 21% of males [46]. Imaging is required to confirm the diagnosis; however, the absence of symptoms and normal urinalysis may be sufficient to exclude prostatic involvement [47]. Drainage of prostatic abscesses is usually required to hasten cure and prevent relapse.

Osteomyelitis and septic arthritis occurs in up to 16% of Australian presentations [17]. Operative intervention is often required, with the majority of patients requiring multiple procedures [48].

Paediatric melioidosis is uncommon in Australia and, in Darwin, has traditionally been associated with skin and soft tissue infection [49,50]. In contrast to Southeast Asia, acute suppurative parotitis is extremely rare in Australia [5], which is likely related to ingestion being an uncommon mode of transmission. Case-fatality rates in Darwin children are comparable to that of adults but, in FNQ, the rates of bacteraemia and mortality are much higher—60% and 50%, respectively—with fatal cases occurring despite optimal treatment [51,52]. The explanation for this observation is uncertain, although it may be partly explained by less active case finding in FNQ. Children in Darwin who present with only skin lesions have been shown to sometimes be infected with minority strains that lack the virulence factor filamentous hemagglutinin gene—fhaB3—which may be more common in FNQ strains [53].

Neurological melioidosis (meningoencephalitis and involvement of the brainstem, cerebellum, and spinal cord) occurs in up to 5% of Australian melioidosis cases [54]. *B. pseudomallei* isolates possessing a *B. mallei*—like bimA allele (*bim_Bm_*) have been shown to have increased persistence in phagocytic cells, increased virulence, and to be neurotropic and, hence, strongly associated with neurological disease [55,56].

**Figure 1:**
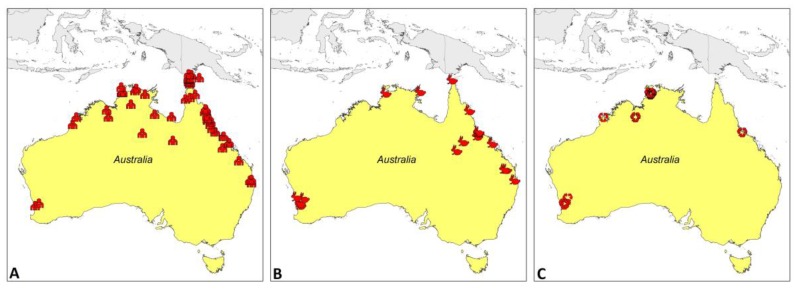
The geographic distribution of confirmed human cases (**A**), animal cases (**B**), and environmental isolates (**C**) of culture-confirmed *B. pseudomallei.*

Environmental sampling has identified *B. pseudomallei* from soil and water across northern Australia [57,58]. *B. pseudomallei* has been isolated from groundwater seeps in endemic areas [59] and melioidosis cases have been linked to contaminated drinking well water [60], as well as two documented outbreaks in remote indigenous communities being linked to contaminated water supplies [36,37]. *B. pseudomallei* is seen in undisturbed, heavily grassed areas, but is also associated with the presence of livestock animals [61]. Soil texture and lower pH levels promote growth of the organism [61]. In Australia, soil exposure often occurs in domestic gardens, [6] where, due to the addition of specific fertilisers and imported grasses, more *B. pseudomallei* is seen compared to other environments [61,62]. *B. pseudomallei* has also been isolated from air samples from outside the home of a patient with suspected inhalational melioidosis [63]. Outbreaks have been associated with contaminated wound irrigation fluid, although this is exceptionally rare [64]. Geographical locations of confirmed cases are shown in Figure 1.

Mapping shows geographical locations of culture-confirmed *B. pseudomallei* isolates only and does not reflect incidence.

## 3. Surveillance Systems and Reporting 

Human melioidosis is a notifiable disease in the NT, Western Australia and Queensland. Notification usually occurs directly from the microbiology laboratory and public health departments gather information about each case, including occupational or recreational exposure, risk factors, and clinical presentation [7]. In the NT, detailed clinical information has been collected prospectively for over 28 years and a similar database has recently been established in Far North Queensland. Guidelines for the management of animal melioidosis are available, including recommendations for safe disposal of dead affected animals [65]. 

## 4. Diagnosis 

Clinicians in northern Australia have a high index of suspicion of melioidosis, particularly during the wet season and in people with predisposing risk factors. Culture, the mainstay of diagnosis of melioidosis, is accessible throughout endemic areas of Australia. Vitek 2 (bioMérieux, France)—the automated biochemical system—is routinely used to confirm the isolation of *B. pseudomallei* from cultured specimens [66].

Matrix-assisted laser desorption/ionization time-of-flight (MALDI-TOF) mass spectrometry is available in many Australian laboratories and can be utilised to rapidly identify *B. pseudomallei* from cultured specimens [67]; however, there is a requirement to expand existing databases with pathogens endemic to different localities to help prevent incorrect identification of other *Burkholderia* species. 

A lateral flow antigen detection assay has been developed as a rapid diagnostic tool, which is proving to be useful as an adjunct to the diagnosis of melioidosis, however, it is not yet routinely available [68]. Polymerase chain reaction is available in some areas to confirm bacterial isolates from clinical specimens, but is not sensitive or specific enough for routine use directly on clinical samples [69,70,71].

Serological testing using an indirect haemagglutination assay is available in Australia, but its use is limited in the diagnosis of acute disease in those from endemic locations because of background seropositivity. It is used for identifying people previously exposed to *B. pseudomallei* prior to commencing immunosuppressive treatment or for seroprevalence surveys [72,73].

In northern Queensland, the microbroth dilution method has been used to provide epidemiological cut-off values of clinical isolates of *B. pseudomallei* to meropenem, ceftazidime, trimethoprim-sulfamethoxazole (TMP-SMX) and doxycycline [74]. Meropenem and ceftazidime provide reliable first-line agents against melioidosis, with primary resistance being extremely rare [75,76]. Primary resistance to TMP-SMX in Australian isolates is also extremely rare [77]. Secondary resistance is also uncommon; however, whole-genome sequencing of *B. pseudomallei* isolates from patients receiving intravenous meropenem for melioidosis treatment have exhibited decreased meropenem susceptibility [78]. Additionally, in cystic fibrosis patients, multidrug-resistant *B. pseudomallei* has been identified after prolonged antibiotic therapy [79]. 

A chest X-ray is performed on all patients with melioidosis. A computed tomography (CT) scan of the abdomen and pelvis is also routinely performed in all adult patients with confirmed or suspected melioidosis to identify abscesses in the prostate, liver, spleen, and kidneys. For pregnant women and children, an abdominal ultrasound is considered a reasonable alternative. In patients with CNS involvement, CT images may be normal and magnetic resonance imaging (MRI) is preferred. MRI often shows extensive hyperintense changes on T2-weighted images; microabscesses, leptomeningeal enhancement, and trigeminal nerve involvement are also common [54,80,81]. 

## 5. Treatment

Treatment of melioidosis in Australia consists of an intensive phase with intravenous antibiotics followed by a prolonged eradication phase with oral antibiotics. Meropenem or ceftazidime is used in the intensive phase and both are readily available in Australia, even in remote areas. Additionally, sick patients from rural or remote areas are quickly transferred to referral hospitals or tertiary care centres. Antibiotic regimens are presented in Table 1. 

In people with neurological infection, bone or joint infection, genitourinary infection, and skin and soft tissue infections, TMP-SMX is added to the intensive regimen during the intensive phase. Folic acid 5 mg (child: 0.1 mg/kg up to 5 mg) orally, daily, is given to all people receiving TMP-SMX.

Due to concerns regarding adherence to prolonged oral antibiotics, in the NT a longer intensive phase has been developed [10] (Table 2). Using this strategy, the rates of relapse in the NT are very low, even when there is poor adherence to oral eradication therapy [6,53]. A prolonged intensive phase is also used in FNQ, although in this region relapse is more commonly explained by poor initial source control rather than non-adherence to oral eradication therapy [17].

To facilitate adherence to prolonged intravenous antibiotic therapy, ceftazidime administered to outpatients via a peripherally-inserted central catheter using 12-h elastomeric infusers has been widely adopted in Australia and is safe and effective [82].

Oral eradication therapy is routinely prescribed and TMP-SMX remains the treatment of choice. Oral doxycycline is considered as an alternative for people who cannot tolerate TMP-SMX, while oral amoxicillin-clavulanate may be used as an alternative in pregnant women or young children [83].

Case-fatality rates have decreased dramatically over the last 20 years, and is felt to be due to earlier recognition and diagnosis, ready access to therapy, and improving intensive care unit support [6,17]. Between 1989–1997, up to 92% of critically unwell patients with melioidosis died; however, with the introduction of an intensivist-led model of care and the empirical use of meropenem, case-fatality rates in this patient group dropped to 26% [84]. The reduced case-fatality rate coincided with the introduction of adjunctive treatment with granulocyte colony-stimulating factor (G-CSF); however, the attributable mortality benefit of G-CSF is uncertain [85,86]. In FNQ, G-CSF is not used and mortality rates are comparable to those seen in the NT [17]. 

## 6. Awareness and Prevention of Melioidosis 

People who are at risk of acquiring melioidosis are advised to wear gardening gloves and footwear when coming into contact with soil [73]. Australian Government authorities provide easily-accessible recommendations on how to prevent melioidosis. This includes advice to remain indoors during wet and windy weather conditions, to wear a mask when using a pressure hose outside and to limit alcohol consumption [87]. A melioidosis awareness campaign in the NT, promulgating the aforementioned recommendations, provided factsheets, posters, and radio announcements to people at risk of acquiring melioidosis, as well as enclosed shoes to particularly vulnerable populations [88].

People receiving long-term haemodialysis are at particular risk of developing melioidosis [89]. In Darwin, prophylaxis using oral TMP-SMX three times per week post-dialysis is a safe and effective way to reduce this risk and is recommended in other areas where melioidosis is prevalent [90]. Melioidosis serology is recommended in all people prior to starting immunosuppressive treatment in the Top End of the NT. If positive, a urine specimen, sputum sample, and throat, rectum and wound swabs should be collected for melioidosis culture to exclude active disease [73]. High-risk immunosuppressed patients are given prophylaxis with daily dosing of oral TMP-SMX for the duration of the wet season. 

In animals, strategies to reduce the risk of acquiring disease include limiting their access to high-risk areas and providing sufficient drainage to help avoid surface water accumulation. It is recommended that penned animals are kept on dry solid ground or concrete [91], while unpenned animals should be removed from the area of contamination source and have their water supplies chlorinated [26]. Ultraviolet light sterilisation may also be used to reduce *B. pseudomallei* levels in contaminated well water [60].

## 7. Major Achievements 

The Darwin Prospective Melioidosis Study started on 1 October 1989. Since this time, the programme has documented the many and varied presentations of melioidosis. The program has, in close collaboration with colleagues in Thailand, also undertaken long-term studies, and developed the current treatment guidelines that are used globally, which have helped halve the case fatality rate of melioidosis in northern Australia from 30% to under 15%. Discoveries include the documentation of the spread of melioidosis by introduced grasses and birds. The storage of all isolates with linked patient data will serve as a resource for future research into diagnostics, therapeutics, and vaccines. 

Over 1000 *B. pseudomallei* isolates from humans, animals, and the environment across Australia have been submitted to the *B. pseudomallei* MLST database (http://bpseudomallei.mlst.net/). This has permitted the comparison between clinical and environmental isolates, as well as studies of the diversity of *B. pseudomallei* in Australia [92].

## 8. Current and Future Challenges 

Changes in the world′s climate is likely to have an effect on the range and transmission of *B. pseudomallei*; a rise in the sea surface and ambient temperature may lead to an increase in melioidosis cases in Australia [40]. The genetic diversity of *B. pseudomallei* populations has been characterised by using multilocus sequence typing (MLST); however, due to its high recombination rate, *B. pseudomallei* isolates may share the same MLST despite being genetically and geographically distinct [93]. Therefore, whole-genome sequencing—which may not always be readily available—is required to identify strain origin in cases where the same ST is identified between geographically-different locations. Geographically-distinct virulence-associated genes have been found to be over-represented in Australian *B. pseudomallei* isolates [94]. The pathogenic nature of most of these virulence factors is not well established and requires further elucidation. 

In addition to environmental factors, host factors, including increasing rates of diabetes mellitus and ongoing hazardous alcohol use are expected to increase the population at risk of melioidosis. Further education targeting risk factors and strategies to prevent *B. pseudomallei* exposure are required. In the absence of a suitable vaccine candidate, the use of prophylactic antibiotics to target at-risk populations requires further research.

Extending the intensive phase of intravenous antibiotics in melioidosis has been associated with favourable outcomes and a minimal risk of relapse; therefore, the benefits of, and ongoing need for, prolonged oral antibiotics during the eradication phase for every patient with melioidosis requires further work. The effect of adequate source control on the duration of therapy also requires exploration.

The above challenges will be made possible with continued collaboration both across endemic areas of Australia, as well as with colleagues internationally. 

## Figures and Tables

**Table 1:** Antibiotic dosing for treatment of melioidosis in Australia.

Phase	Antibiotic	Adult Dose	Child Dose
Intensive	Meropenem	1 g intravenously 8-hourly 2 g intravenously 8-hourly ^1^	25 mg/kg up to 1 g intravenously 8-hourly 50 mg/kg up to 2 g intravenously 8-hourly ^1^
Intensive	Ceftazidime	2 g intravenously 6-hourly	50 mg/kg up to 2 g intravenously 6-hourly
Intensive and eradication	Trimethoprim-sulfamethoxazole	≥60 kg: 320 + 1600 mg orally 12-hourly 40–60 kg: 240 + 1200 mg orally 12-hourly	6 + 30 mg/kg up to 240 + 1200 mg orally 12-hourly
Eradication	Amoxicillin-clavulanate	20/5 mg/kg orally 8-hourly	20/5 mg/kg orally 8-hourly
Eradication	Doxycycline	100 mg orally 12-hourly	Not recommended

^1^ For cases with neurological involvement.

**Table 2:** Recommended antibiotic duration for the treatment of melioidosis in Australia.

Site of Infection	Minimum Intensive Phase Duration (Weeks)	Eradication Phase Duration (Months)
Cutaneous infection only	2	3
Bacteraemia without focus	2	3
Pneumonia without lymphadenopathy or ICU admission	2	3
Pneumonia with lymphadenopathy ^1^ or ICU admission	4	3
Deep seated collection ^2^	4 ^3^	3
Septic arthritis	4 ^3^	3
Osteomyelitis	6	6
Central nervous system infection	8	6
Mycotic aneurysm or other arterial infection	8	6

^1^ Defined as any hilar or mediastinal lymph node greater than 10 mm. ^2^ Involving the liver, spleen, kidneys, or prostate. ^3^ Start of intensive phase begins after the last drainage or tissue specimen grows *B. pseudomallei*.
